# Endoureteral Management of Renal Graft Ureteral Stenosis by the Use of Long-Term Metal Stent: An Appealing Treatment Option

**DOI:** 10.1089/cren.2016.0084

**Published:** 2016-09-01

**Authors:** Patrick-Julien Treacy, Art R. Rastinehad, Laetitia Imbert de la Phalecque, Laetitia Albano, Matthieu Durand

**Affiliations:** ^1^ Department of Urology, Hôpital Pasteur 2, Nice Sophia-Antipolis University, France.; ^2^ Department of Urology, MSSM, New York City, New York.; ^3^ Department of Kidney Transplantation, Hôpital Pasteur 2, Nice Sophia-Antipolis University, France.; ^4^ INSERM, U1189, ONCO-THAI, Lille, France.

**Keywords:** imaging, long-term metal stent, renal graft, ureteral stricture

## Abstract

***Background:*** Ureteral stenosis is part of the common complications of renal graft reported in 3% to 7% of cases. Multiple treatments have been introduced regarding length and position of the stenosis. Metal stents for urologic purpose were created in 1998. Double percutaneous antegrade and transurethral retrograde access to a ureteral stenosis to a long-term metal stent procedure has been rarely described.

***Case Presentation:*** Here, we present a case of a ureteral stricture in a double ipsilateral kidney graft with a common ureter. A 67-year-old patient presented with obstructive nephritis associated with acute renal failure 6 years after a double renal graft with a uretero-ureteral end-to-side anastomosis. Abdominal CT scan showed double pelvic dilation. The patient underwent double percutaneous nephrostomies and antegrade pyelogram showed both renal pelvic and ureter dilations caused by a severe chronic ureteral stenosis at junction into the bladder. A Double-J ureteric stent was then inserted retrogradely over a guidewire as first-line treatment. Due to recurrent urinary tract infections (UTIs), removal and replacement of Double-J stents were carried out by placing a thermoexpandable metal stent Memokath^®^ 051 (Bard, Pnn Medical) through the common ureter by a double antegrade and retrograde approach. Treatment was effective with a good renal function maintained after a 3-year follow-up without UTIs.

***Conclusion:*** Double antegrade and retrograde access to a long-term metal stent treatment can be seen as an alternative treatment to either endoscopy or open surgery. Further studies should be continued using larger series.

## Introduction

Ureteral stenosis is part of the common complications of renal graft reported in 3% to 7% of cases. This includes various etiologies of both intrinsic and extrinsic ureteral diseases at different locations, the management of which is always challenging. Various techniques have been performed to treat these strictures, including percutaneous, endoscopic, or open surgery approaches. First cases of metal stent used in kidney graft stenosis were done in 2005.^1^ We report here the use of endoureteral metal stent by using both antegrade and retrograde approaches in the management of a complex ureteral stenosis in kidney graft.^2^ Written informed consent was obtained from the patient for publication of images.

## Background

Initially, in 1998, this 67-year-old patient with an undetermined renal failure underwent a double ipsilateral renal graft using a uretero-ureteral end-to-side anastomosis(UUA) of the inferior kidney transplant into the upper one, ending in a single ureterovesical anastomosis. He had a history of long-term hemodialysis before transplantation, accompanied by complete anuria and small capacity of the bladder. Kidney transplantation was effective: serum creatinine decreased and patient was discharged from the hospital 10 days after surgery. Allograft function was maintained well overtime. In the next 6 years, the patient did not experience any complication including hydronephrosis in the graft, urine extravasation, or urinary tract infection (UTI).

In 2004, an ultrasound (US) scan first showed a 14-mm dilation of the renal pelvis on the superior graft, with no dilation reported on the inferior graft. During follow-up 1 year later, pelvic dilation was reported on both grafts, with a 14- and 13-mm dilation on the superior and inferior grafts, respectively. Renal function was not affected, with a GFR of 92 mL/min up to 2009, but in 2010, the GFR started to decrease at 71 mL/min, as dilation increased to 26 mm and 20 mm on the superior and inferior graft, respectively. In September 2013, the patient was admitted to the Kidney Transplantation Unit with hyperthermia associated with anuria and elevated serum creatinine (212 μM). US scan showed a major ureterohydronephrosis with larger pelvic dilation of, respectively, 30 and 26 mm.

## Treatments

**FIG. 1.**
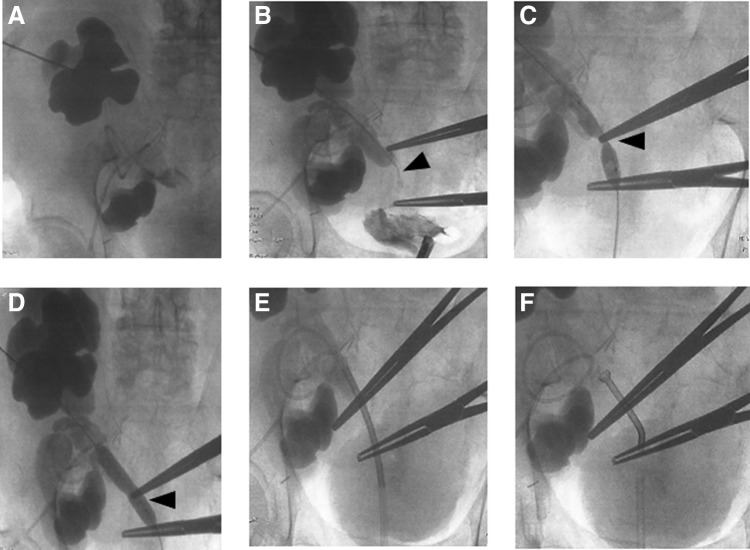
Thermoexpandable metal stent placement on a ureteral stenosis. This figure show obstructive pyelonephritis in a 67-year-old patient with a double renal graft and a uretero-ureteral end-to-side anastomosis. A puncture of the superior graft was carried out demonstrating a major renal pelvic dilation of the graft on antegrade pyelogram **(A)**. Fifteen minutes later, **(B)** a double renal pelvic dilation associated with a ureteral dilation was noted above the severe ureteral stenosis on the common iliac ureter (*arrowhead*). A 0.035 Terumo^®^ guidewire was placed through the stenosis into the bladder. Through the wire, a dilatation of the ureteral stenosis was performed with a 5 mm high pressure (at 10 atm) dilator **(C)**, with effectiveness **(D)** (*arrowheads*). Then, a cystosocopy collected the wire by a retrograde approach. After dilatation, an access sheath was left on the stenosis, through a retrograde approach, allowing the correct placement of Memokath^®^ 051 over the stenosis **(E)**. An effective expansion of the metal stent was achieved after removal of the sheath using 60°C hot water **(F)**.

The patient underwent double percutaneous nephrostomies in the grafts and was effectively cured by antibiotics. One week later, an antegrade pyelogram showed both major renal pelvic and ureter dilations caused by a severe ureteral stricture at the junction of the common ureter into the bladder (Fig. 1A). One 6/16 Double-J stent was temporarily passed over a guidewire from the inferior graft through the stenosis. Although this was initially effective, overtime, multiple acute UTIs were reported with rising serum creatinine.

As a consequence, the Double-J stent was retrieved. A puncture of the superior kidney graft was carried out, showing a major renal pelvic dilation of the graft under general anesthesia. After 15 minutes, opacification demonstrated double pelvic dilation associated with a ureteral dilation among a major stenosis of the common ureter (Fig. 1B). Subsequently, a straight 0.035F TERUMO^®^ guidewire was passed through the nephrostomy, permitting retrograde access to the stenosis into the bladder. A dilatation was then performed using a 5-mm high pressure dilator (10 atm), using a radiopaque landmark to point out the location of the stenosis to place the balloon (Fig. 1C). By carefully inflating the balloon, the stricture was gradually dilated effectively in percutaneous antegrade manner to permit the passageway (Fig. 1D).

Once the pathway was established, a cystoscopy was carried out to collect the wire, allowing retrograde access to the stent placement location. A single stent was chosen rather than tandem stents in this first stenting approach on a nonmalignant stenosis. An access sheath was then left on the stenosis with a retrograde approach (Fig. 1E). The insertion system was introduced with the ureteral stent into the access sheath to place it over the stenosis. After removal of the access sheath, 20 mL of 65°C sterile water was injected into the insertion system to fully expand a 60-mm thermo-expandable metal stent Memokath^®^ 051 (PNN Medical, Copenhagen, Denmark). Placement was effective, and patency of the stent was controlled by multiple parameters, such as US scan (absence of pelvic dilation), diuresis, and serum creatinine, 2 weeks after surgery, then on postoperative consultations at M1 and M3 after surgery. One week after surgery, blood tests showed a major decrease of serum creatinine and US scan showed a complete restriction of the pelvic dilation.

After a 3-year follow-up, the patient had not experienced any treatment-related complications, and controll imaging showed the original stent still at its initial place.

## Discussion

This case highlights the use of endoureteral stent in the management of complex ureteral stenosis and demonstrates that it could be an alternative in the therapeutic armamentarium. For instance, it outlines the treatment options to cure distal ureteral strictures. He et al. described three types of stenoses, according to different specificities, such as hydronephrosis, stricture on pyelogram, and associated lymphocele, and concluded with a specific treatment for each type of stenoses.^3^ In such circumstances, the optimal imaging for defining the extent of the ureteral stricture is an antegrade pyelogram, which provides relevant images to distinguish the various locations of the stenosis. Exhaustive and thorough information is needed to determine the best option for repair, whether it be an endourologic or a minimally invasive approach, or open surgical techniques.

The most common initial management of a benign ureteral stricture is balloon dilatation, followed by stent placement for 4–6 weeks. Hafez and Wolf reviewed eight published series of ureteral stenoses managed with balloon dilatation.^4^ Success rates ranged from 48% to 88%. Out of the 280 ureteral stenoses treated, the overall mean success rate was 55%. They found that balloon dilatation was best suited for very short nonischemic strictures.

In addition, Goldfischer and Gerber summarized the results of balloon dilatation in a large series and found this procedure to yield a success rate of 50%–76%.^5^ Factors associated with a good outcome included short duration (<3 months) and short length of stenosis. Given the frequent need for multiple procedures and the higher success rate associated with endoureterotomy, most urologists recommend endoscopic incision as the initial minimally invasive management of ureteral stricture disease.^6^

In our case, Double-J stenting failed because of recurrent ureteral stricture. Metal stents have often been described as the evolution of Double-J stenting, with the theoretical advantages that they bring, including reduced encrustation, improved tensile strength and stability, prolonged stent indwell time, and better flow. Maan et al. also showed the benefits of Memokath stents in terms of quality of life, lower urinary tract symptoms, or even on chronic pain, with surprising results.^7^

In such a complex case with two ureters combined with UUA in a common distal stenosis ureter after more than 15 years of antirejection therapy, open surgery for ureteral reconstructions is very likely to lead to graft transplantectomy. All open procedures carry an increased risk of morbidity, increased recovery time, and increased length of hospitalization than endoscopic approaches, with a global success rate of 60% to 72% according to a review of published literature.^8^

Metal stents have been used to treat end-stage malignant disease. Stent removal is reported to be extremely difficult at first and stent migration is frequent. Moskovitz and colleagues observed a migration in more than 14% of the stents placed, mandating a stent removal.^9^ Owing to innovation in the materials and design of ureteral stents, this is likely to evolve. The development of allium stents is the most recent advancement in stenting development, with great effectiveness in several studies.^10,11^ Given that only a limited prospective cohort study has been carried out so far, it is still extremely difficult to assess this technique. However, there have been some attempts to apply them to benign ureteral strictures, ureteropelvic junction obstruction, and ureterovesical obstruction. Liatsikos et al. reported a total of 142 ureters stented in 102 patients.^12^ The primary stent patency rate was 66%. Papatsoris and coworkers also revealed great results, concerning both benign and malignant diseases, with only six treatment failures out of 86 stent placements after a mean follow-up period of 17.1 months.^13^

This could very certainly become an appealing option to preserve renal function when ureteral stenosis cannot be managed by long-term Double-J stenting.

## Conclusion

Few cases of long-term metal stent placement as a treatment of renal graft ureteral stricture have been reported so far, especially in a double ipsilateral graft with a UUA. The double percutaneous antegrade and transurethral access to the stricture was a compelling approach for such a challenging situation.

## Abbreviations Used

CT: computed tomography
GFR: glomerular filtration rate
UUA: uretero-ureteral end-to-side anastomosis
UTIs: urinary tract infections
US: ultrasound
